# Diagnostic challenge in veterinary pathology: Otitis in a humanized NOG-EXL mouse

**DOI:** 10.1177/03009858241279141

**Published:** 2024-09-21

**Authors:** Elinor Willis, Jillian Verrelle, Anthony Secreto, Stephen D. Cole, George McClung, Kelley M. Weinfurtner, Terence P.F. Gade, Enrico Radaelli

**Affiliations:** 1 Department of Pathobiology, School of Veterinary Medicine, University of Pennsylvania, Philadelphia, PA; 2 Stem Cell and Xenograft Core, Perelman School of Medicine, University of Pennsylvania, Philadelphia, PA; 3 Penn Image-Guided Interventions, Department of Radiology, Hospital of the University of Pennsylvania, Philadelphia, PA; 4 Division of Gastroenterology and Hepatology, Department of Medicine, Hospital of the University of Pennsylvania, Philadelphia, PA; 5 Department of Cancer Biology, Perelman School of Medicine, University of Pennsylvania, Philadelphia, PA; 6 Department of Radiology, Corporal Michael J. Crescenz Philadelphia VA Medical Center, Philadelphia, PA

## Clinical History and Gross Findings

A 33-week-old, female, NOD.Cg-*Prkdc*^*scid*^ *Il2rgtm1Sug* Tg(SV40/HTLV-IL3,CSF2)10-7Jic/JicTac (NOG-EXL) mouse was humanely euthanized due to neurologic signs, including twitching, head tilt, and circling. At 10 weeks of age, this mouse had been conditioned with busulfan and then received 1 × 10^5^ human adult bone marrow-derived CD34^+^ hematopoietic stem and progenitor cells 24 hours later. Two weeks after humanization, a patient-derived hepatocellular carcinoma was implanted orthotopically in the liver.

At necropsy, the mouse had poor body condition as well as moderate splenomegaly. The liver had an approximately 5 mm diameter tan nodule in the left lobe. The ventrolateral head, in the region of the right tympanic bulla, was unilaterally expanded by a firm, tan, approximately 3 mm diameter mass.

## Microscopic Findings

**Figure 1.**
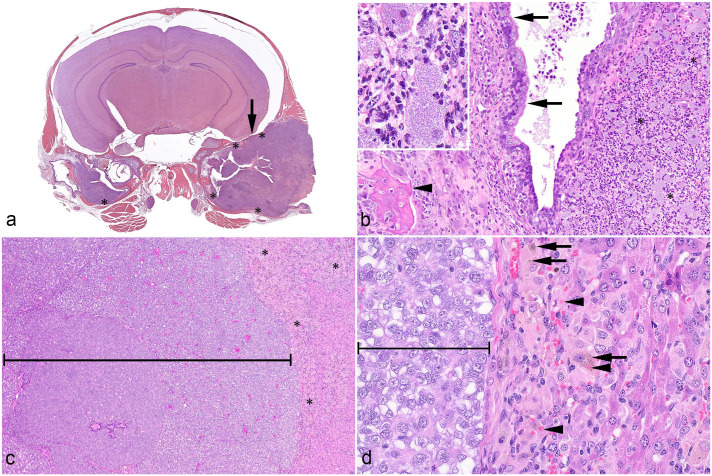
Hematoxylin and eosin. (a) Cross section of the head. The tympanic cavities are filled by suppurative inflammation with multifocal bone lysis (*) and focal meningeal involvement (arrow). (b) Higher magnification of the middle ear with suppurative inflammation (*), including large macrophages, mucoperiosteum (arrows) elevated by inflammatory cells and granulation tissue, and bone lysis (arrowhead). Inset: suppurative inflammation including large macrophages with numerous intracytoplasmic bacteria. (c) Liver with patient-derived, experimentally implanted, solid hepatocellular carcinoma (bracket), and infiltrating inflammatory cells (*). (d) Higher magnification of the hepatic lesions showing hemophagocytic infiltrates of macrophages and multinucleated cells containing hemosiderin (arrow) and intact erythrocytes (arrowhead) adjacent to carcinoma (bracket).

Bilaterally, with the right side more severely affected, the tympanic cavity was filled by suppurative inflammation characterized by a dense infiltrate of degenerated neutrophils and macrophages admixed with necrotic debris (Fig. 1a, b). The right tympanic membrane was ruptured. The mucoperiosteum was expanded by a similar inflammatory infiltrate as well as fewer lymphocytes, plasma cells, granulation tissue, and edema. The adjacent petrous temporal bone and calvaria exhibited osteolysis with the inflammation unilaterally extending into the meninges (Fig. 1a, b). Intralesional macrophages were severely distended by innumerable, intracytoplasmic, rod-shaped bacteria (Fig. 1b, inset).

The liver had a focal patient-derived, experimentally implanted, solid hepatocellular carcinoma (Fig. 1c, d). The peritumoral hepatic lobules had a moderate multifocal infiltrate of large macrophages and multinucleated giant cells with abundant eosinophilic cytoplasm that often contained hemosiderin and/or intact erythrocytes (Fig. 1d). There was also a mild portal infiltrate of lymphocytes and plasma cells, as well as multiple small foci of hematopoietic precursors (extramedullary hematopoiesis). In addition, occasional individual hepatocyte degeneration and death were observed. In the lungs, pulmonary veins had mild, mixed granulocytic and mononuclear to granulomatous infiltrates that elevated the endothelium or expanded the adventitia. The lymphoid tissues lacked normal architecture. The splenic white pulp was expanded by mononuclear cells with large macrophages and fewer multinucleated giant cells that occasionally contained hemosiderin. The red pulp had mild extramedullary hematopoiesis and rare fibrin thrombi. Lymph nodes contained a similar mononuclear cell infiltrate with large macrophages. In the bone marrow, normal hematopoietic cells were displaced by multiple clusters of macrophages and multinucleated giant cells, as well as an increased population of eosinophil precursors with distinct cytoplasmic granules.

Additional findings in this mouse included mild vacuolation of the brainstem and spinal cord (a common background finding in this strain^4^) and minimal degeneration of the Harderian gland.

## Differential Diagnoses

Common bacterial causes for otitis media in mice include *Klebsiella* spp., *Rodentibacter pneumotropicus* (formerly *Pasteurella pneumotropica*), *Pseudomonas aeruginosa*, *Burkholderia* spp., and *Mycoplasma pulmonis.*^8^ Predisposing infections, such as with Sendai virus or *Mycoplasma* spp., should also be considered.^8^ In the experimental context, mice may also be used as models for otitis media, and the most commonly used pathogens include *Streptococcus pneumoniae*, *Haemophilus influenzae*, and *Moraxella catarrhalis.*^3^

In addition to the otitis media with otitis externa and interna, osteolysis, and meningitis, this mouse had multiorgan histiocytic to granulomatous inflammation. Given the severity of the otitis media with local extension and the profound immunodeficient status conferred by the NOG-EXL background, septic complication with secondary systemic involvement was considered possible. Moreover, this mouse was humanized with CD34^+^ hematopoietic stem and progenitor cells and the NOG-EXL background also includes transgenic expression of human myeloid stimulatory cytokines. Therefore, chimeric myeloid cell hyperactivation syndrome (a complex phenotype that encompasses macrophage activation syndrome or hemophagocytic lymphohistiocytosis-like disease, as well as hyperproliferation of other myeloid lineages) should be included in the differential diagnosis, especially because of the evidence of erythrophagocytosis and the increased eosinophilopoiesis.^6,10,11^

## Further Investigations and Diagnosis

**Figure 2.**
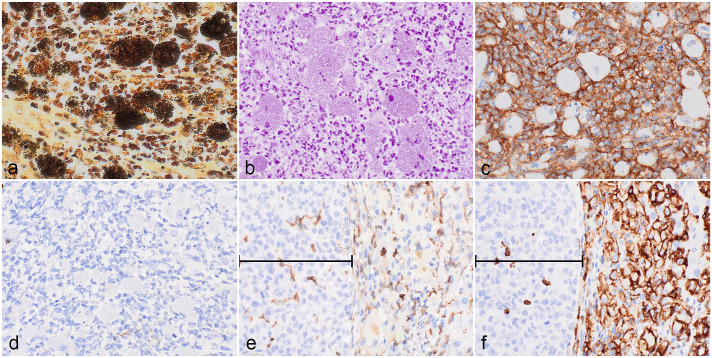
(a-d) Inflammatory infiltrate in the tympanic cavity. (a) Numerous short rods within macrophages and neutrophils, as well as fewer extracellular bacteria. Warthin-Starry stain. (b) Intrahistiocytic bacteria are gram-negative. Gram stain MacCallum-Goodpasture method. (c and d) The large macrophages containing bacteria exhibit positive immunolabeling for mouse CD45 (c) and are negative for human CD45 (d). (e and f) Infiltrate in liver. Large macrophages and multinucleated giant cells are negative for mouse CD45 (e) and positive for human CD45 (f). Bracket denotes patient-derived solid hepatocellular carcinoma.

The bacteria observed in association with the otitis media, otitis interna, otitis externa, osteolysis, and meningitis were Warthin-Starry-positive (Fig. 2a) and gram-negative (Fig. 2b). Warthin-Starry-positive bacteria were not observed in association with macrophages and multinucleated giant cells infiltrating the spleen, liver, lymph nodes, lungs, and bone marrow. Aerobic bacterial culture performed on several NOG-EXL mice with similar lesions within the same colony and on swabs from the cage where the animal was housed revealed pure growth of the bacterium *Burkholderia multivorans* as identified by matrix-assisted light desorption/ionization-time of flight analysis on the Bruker Biotyper Sirius. Given that a culture was not carried out on this specific animal, conventional polymerase chain reaction (PCR) was performed on formalin-fixed paraffin-embedded tissue from the middle ear lesions as previously described.^1^ The PCR was positive for *Burkholderia* spp. Altogether, the results of these additional investigations support the diagnosis of severe bilateral suppurative otitis media with local extension due to opportunistic *B multivorans* infection.

Immunohistochemistry for human- and mouse-specific CD45 was performed on the ear, liver, spleen, lymph nodes, lungs, and bone marrow. Greater than 90% of the inflammatory cells in the middle ear lesion, including the large rod-shaped bacteria-laden macrophages, exhibited strong membranous immunolabeling for mouse-specific CD45 (msCD45; Fig. 2c). Scattered small mononuclear cells were positive for human-specific CD45 (huCD45; Fig. 2d). In contrast, the infiltrating macrophages and multinucleated giant cells in the liver, spleen, lymph nodes, lungs, and bone marrow were negative for msCD45 (Fig. 2e) and positive for huCD45 (Fig. 2f). The msCD45+ cells in the liver were interpreted as Kupffer cells and a few small mononuclear cells infiltrating within and adjacent to the tumor. Scattered small mononuclear huCD45+ cells also infiltrated the tumor. Because of the different mouse and human origins of the macrophages observed in the otitis and other affected organs, respectively, the systemic histiocytic to granulomatous infiltrate is unlikely to result from the septic spread of the primary ear infection. In addition, eosinophilic precursors in the bone marrow had distinct cytoplasmic granules consistent with human morphology. In this context, the predominance of hemophagocytic huCD45+ macrophages in the liver, in combination with extensive human eosinophilopoiesis, is most indicative of myeloid cell hyperactivation syndrome^11^.

## Discussion

*Burkholderia multivorans* is a member of the *Burkholderia cepacia* complex, which is a group of opportunistic pathogens known to cause pneumonia in cystic fibrosis patients and contaminate medical products.^9^ The closely related species *Burkholderia gladioli* has been reported as a cause of otitis media in immunodeficient mice.^2,5^

In the case presented here, activated macrophages were found in the middle ear lesion and systemically in the liver, bone marrow, lungs, spleen, and lymph nodes. While systemic lesions were reported in mice with *B gladioli* infection, neutrophils were the prominent immune cells rather than macrophages.^2,5^ The predominance of murine macrophages in the middle ear and human macrophages in the other affected organs also indicates the occurrence of two separate processes rather than systemic consequences of the otitis media.

*Burkholderia* spp. can cause fatal infections in human patients with chronic granulomatous disease, a disorder in which phagocytes cannot generate reactive oxygen species to kill infectious organisms.^9^ In the examined humanized mouse, human macrophages did not respond to the infection despite exhibiting evidence of proliferation and systemic hyperactivation. Murine macrophages did respond, although the massive expansion of both intra- and extracellular bacteria suggests impaired killing function. Interestingly, functions of phagocytic cells in NOG and NSG mice are likely compromised as their NOD background is associated with defects in antigen presentation, C5 complement function, and defective interleukin-1 (IL-1) secretion in lipopolysaccharide-activated macrophages.

Chimeric myeloid cell hyperactivation syndrome has been described in humanized NSG-SGM3 (NOD.Cg-*Prkdc*^*scid*^ *Il2rg*^*tm1Wjl*^ Tg(CMV-IL3,CSF2,KITLG)1Eav/MloySzJ)^6,10^ and more recently NOG-EXL mice^11^ and is characterized by multiorgan infiltrates of hemophagocytic histiocytes, anemia, increased eosinophilopoiesis, and, in NSG-SGM3 mice, mastocytic infiltration of the pancreas. Myeloid cell hyperactivation syndrome often limits the lifespan and, thus, the utility of these mice. The excessive activation and proliferation of human myeloid cells is likely due to the supraphysiologic expression of human myeloid stimulatory cytokines, namely granulocyte-macrophage colony-stimulating factor and IL-3 (both strains) and KIT ligand (NSG-SGM3 only).^7^

In summary, this diagnostic case emphasizes the importance of recognizing unintended consequences of CD34+ hematopoietic stem and progenitor cells humanization in severely immunodeficient mice and discriminating them from opportunistic infections caused by unconventional microbes that are usually nonpathogenic in immunocompetent hosts.
